# 24-hour Movement Questionnaire (QMov24h) for adults: development process and measurement properties

**DOI:** 10.1186/s12966-024-01667-7

**Published:** 2024-10-09

**Authors:** Bruno Rodrigues, Pedro B. Júdice, Adilson Marques, Eliana V. Carraça, Luís Lopes, Eduarda Sousa-Sá, Jorge Encantado, António Videira-Silva, Dylan P. Cliff, Romeu Mendes, Rute Santos, on behalf of the QMov24h working group, Analiza M. Silva, António L. Palmeira, Aristides M. Machado-Rodrigues, Arnaldina Sampaio, Carla Moreira, César Agostinis-Sobrinho, Diogo Lima, Filipe Biscoito, João Rocha, Raul Martins, Sandra Abreu, Susana Vale, Teresa Figueiras, Teresa Pereira, Vera Simões

**Affiliations:** 1 Research Centre in Physical Activity, Health and Leisure, Faculty of Sport, University of Porto, Porto, Portugal; 2 Escola Superior de Desporto de Rio Maior, Instituto Politécnico de Santarém, Rio Maior, Portugal; 3 Programa Nacional para a Promoção de Atividade Física, Direção-Geral da Saúde, Lisboa, Portugal; 4 Faculdade de Educação Física e Desporto, CIDEFES, Universidade Lusófona, Lisboa & CIFI2D, Universidade do Porto, Porto, Lisboa Portugal; 5 CIPER, Faculdade de Motricidade Humana, Lisboa, Portugal; 6 Laboratory for Integrative and Translational Research in Population Health, Porto, Portugal; 7 Early Start, School of Education, Faculty of the Arts, Social Sciences and Humanities, University of Wollongong, Wollongong, NSW Australia; 8 EPIUnit-Instituto de Saúde Pública, Universidade do Porto, Porto, Portugal; 9 Unidade Local de Saúde de Trás-os-Montes e Alto Douro, Vila Real, Portugal; 10 Research Centre on Child Studies, University of Minho, Braga, Portugal; 11 Institute of Education, University of Minho, Braga, Portugal

**Keywords:** Movement behaviours, Questionnaires, Monitoring and surveillance, Validation, Adults

## Abstract

**Background:**

Sleep, sedentary behaviour, and physical activity are essential components within the 24-hour time frame. Existing questionnaires used to measure these behaviours have insufficient measurement properties and are unsuitable for assessing compliance with the WHO Physical Activity and 24-hour Movement Guidelines. To describe the development process of the 24-hour Movement Questionnaire (QMov24h) and its testing. The QMov24h was developed to gather detailed information on sleep, sedentary behaviour, and physical activity.

**Methods:**

The sample comprised 117 participants (58% women), aged 30.95 ± 13.56 years. The development process of the QMov24h followed the COSMIN guidelines: (i) Construction of items; (ii) Face validity with end-users; (iii) Content validity with experts; (iv) Criterion validity against accelerometry and convergent validity against diary assessments; and (v) 7-day test-retest reliability.

**Results:**

The QMov24h presented adequate content and face validity. The QMov24h showed moderate criterion validity for sleep (rho=0.343;*p*<0.001), light physical activity (rho=0.31;*p*=0.002) and total aerobic physical activity (rho=0.343;*p*<0.001), as well as strong criterion validity for sedentary behaviour (rho=0.428;*p*<0.001) and aerobic moderate-to-vigorous physical activity (rho=0.534;*p*<0.001). Reliability varied from poor to excellent (ICC from 0.38 to 0.962;*p*<0.001) for all questionnaire variables. Regarding compliance of the 24-hour movement guidelines, the questionnaire also showed a strong to almost perfect percentage of agreement with accelerometry (from 69% to 94.3%), and minimal to strong reliability (k from 0.38 to 0.87) between the first and second administrations of the QMov24h.

**Conclusions:**

The QMov24h questionnaire is a valid and reliable tool for assessing levels of movement behaviours and compliance with guidelines in adults. Its measurement properties are comparable to, or even better than, those of existing questionnaires, while posing a similar burden to participants. The QMov24h is useful for research, clinical practice, and public health surveillance. The QMov24h has strong psychometric properties, making it suitable for translation, cultural adaptation, and testing in diverse populations for broader international use.

**Supplementary Information:**

The online version contains supplementary material available at 10.1186/s12966-024-01667-7.

## Background

Sleep, sedentary behaviour (SB), and physical activity (PA) collectively constitute essential components within the 24-hour time frame. These movement behaviours (MovBeh) are time-dependent since any change in one behaviour is necessarily done at the expense of any of the others [1–3]. Recently, some countries have begun to develop 24-hour movement guidelines for adults [4–6], based on evidence that adequate combinations of MovBeh are health-related [2, 3, 7]. Adopting the 24-hour movement guidelines will provide important information for clinicians, researchers, policymakers and the general population, given the synergistic effects of MovBeh on health. However, this could be challenging for public health surveillance systems, given the difficulty in assessing MovBeh in an integrated fashion.

In large epidemiological studies and clinical settings, self-reported instruments are socially acceptable, cost-effective, avoid burdening participants with cumbersome equipment, and have minimal influence over normal PA patterns [8–10]; therefore, they are often chosen over device-based measurements to assess MovBeh. Certainly, questionnaires offer a more practical and simpler way to collect data from large samples, when compared to device-based instruments. Moreover, questionnaires inherently carry a negligible risk of perturbing regular patterns of MovBeh since the designated reference period for measurement typically precedes their administration [11]. However, as recently observed in a systematic review, existing questionnaires to measure sleep, SB and PA show insufficient measurement properties and frequent methodological limitations, and none was developed considering the 24-hour MovBeh paradigm [12]. Likewise, the existing questionnaires do not align with the 24-hour movement guidelines’ paradigm [13]. For example, the International Physical Activity Questionnaire (IPAQ) [14], a widely applied PA questionnaire which was developed to monitor and guide PA policies through comparable data between countries, asks to report only activities lasting more than 10 minutes and does not differentiate muscular strengthening activities from aerobic activities. In contrast, the current PA guidelines of the WHO advocate that “every move counts” and recommends for muscular strengthening activities [15]. Moreover, current questionnaires [14, 16] focus mainly on moderate-to-vigorous PA (MVPA) and there is increasing evidence and interest on the potential health effects of light PA (LPA) [17, 18], that is typically composed of non-structured and incidental activities [19], which are increasingly represented in the new recommendations. Although it is important to have comparable country data over the years through the stability of self-reported instruments, it is of most importance that available instruments provide accurate information allowing assessment of compliance with current guidelines [13]. The development of new questionnaires to access all MovBeh with good measurement properties is challenging, given that there is no single gold standard instrument to measure all MovBeh. Nevertheless, there are procedures that can be employed that may potentially enhance the measurement properties of MovBeh questionnaires. These include (i) applying the best practices of questionnaire development, as outlined in the literature [8–10, 20]; (ii) performing face and content validity processes to ensure that questionnaires are adapted to the study populations and that the questionnaires’ constructs are appropriate [8–10, 20]; (iii) applying appropriate statistical procedures, such as Intraclass Coefficients Correlations, instead of correlations for test-retest reliability [21]; and (iv) ensuring that the instructions of the questionnaire are thorough, so that respondents understand the covered concepts.

In this context, the new 24-hour MovBeh paradigm and guidelines raise the need to develop new questionnaires to accurately assess MovBeh in an integrated manner and adapt monitoring and surveillance systems to assess compliance with the 24-hour movement guidelines and track changes over time. Therefore, in this context, the objective of this study is to describe the development process of the 24-hour Movement Questionnaire (QMov24h), encompassing stages such as item construction, face validity, content validity, criterion validity, convergent validity, and test-retest reliability. The QMov24h was specifically devised to elicit comprehensive information pertaining to sleep, SB, and PA.

## Methods

The present study was conducted following the design and analysis of the Consensus-based Standards for the Selection of Health Measurement Instruments (COSMIN) guideline. The process began by developing the *24-hour Movement Questionnaire* (QMov24h) through a three-stage approach: (i) item construction, (ii) face validity testing with end-users, and (iii) content validity testing by experts. Then, measurement properties of the final version of the questionnaire were assessed (criterion and convergent validity; and test-retest reliability).

Prior to data collection, ethics approval was obtained from the Faculty of Sports’ Ethics Committee of the University of Porto (CEFADE 17 2022). Written informed consent was provided by all participants. The survey’s content and procedures were designed according to the Helsinki Declaration [22].

### Development of the *24-hour Movement Questionnaire* (QMov24h) for adults

#### Questionnaire rationale

The QMov24h aims to measure sleep, SB, and PA in adults over a 24h period, and to be used as a screening tool in clinical settings, observational studies and clinical trials.

Prior to the development of the QMov24h, and based on the findings and gaps identified in a previous review [12], the following needs were outlined: (i) capture sleep duration, SB duration, PA volume (duration and frequency) and intensity (LPA, Moderate PA [MPA], and Vigorous PA [VPA]) by PA type (aerobic and strength), as well as balance activities (i.e., activities that challenge the ability to remain static or those which challenge the ability to balance while moving); (ii) cover important information from a public health and surveillance points of view, namely to assess compliance with current 24-hour movement guidelines; (iii) be adaptable to future changes in the guidelines, i.e., provide continuous outcome variables; (iv) be informative and educational, so that respondents can understand the concepts covered by the questionnaire before they answer it, to minimize response bias; (v) formulate questions that prevent overlap in the reporting of behaviours, i.e., the sum of all questions should roughly account for 24 hours; (vi) avoid considerable burden to participants (less than 15 min of answering time); (vii) allow data analysis from each of the MovBeh, whilst respondents answer the whole questionnaire; (viii) be crafted in a neutral format for versatile applicability across various countries and cultural backgrounds, or readily adaptable to diverse international contexts; and (ix) be answered either with paper and pen, or in digital format.

Item construction

A systematic review [23] was conducted to compile and evaluate the content quality and measurement properties of existing questionnaires measuring MovBeh. This review provided a theoretical and practical background to outline the first QMov24h draft. which was outlined by the authors BR and RS. Throughout three meetings, the authors BR, RS, JE, AM, EVC, ESS, RM and LL, discussed the design, structure and organization of the QMov24h, as well as the definitions and examples included. The order and logic of the questions and item wording were also debated in these meetings.

The first version of the QMov24h comprised 57 items from 13 questions across 6 different domains: sleep (naps and nocturnal sleep); SB (total, during work and out of work); LPA (aerobic and muscular strengthening activities); MPA (aerobic and muscular strengthening activities); VPA (aerobic and muscular strengthening activities); and balance activities.

The recall period of the questionnaire was the usual activity pattern during a typical week, and the chosen response format was hours and minutes per usual day.

Face validity by end-users

Face validity is an informal review of a questionnaire by non-experts to assess its clarity, comprehensibility and appropriateness for the target group [8]. Additionally, testing face validity can identify misperceptions of the text, which may lead to response errors and provide valuable insights into how respondents understand, retrieve and formulate their responses [24]. Face validity was tested on the first version of the QMov24h with a convenient group of 25 participants, using a snowball methodology, that strived to maximise participant variation regarding the PA level, age, gender, literacy, education level and social status. For face validity, cognitive interviews [24] were conducted by experienced interviewers following a semi-structured script to understand end-user’s opinion on the ease of completion and relevance, comprehensiveness and comprehensibility of the instructions, definitions and examples, items and response options, as well as item wording and recall period [8]. These interviews used a think-aloud methodology and verbal probing [9, 25], and were recorded and transcribed for analytic purposes. Then data were independently analysed by content analysis by two authors (BR, JE). After content analysis, data were organized in key themes (e.g., difficulties, misunderstandings, suggestions) and discussed with all authors. The insights gained from the interviews were used to draft the second version of the questionnaire by revising the questionnaire’s design and rewording some parts of the text.

Content validity by experts

Content validity is a qualitative formal assessment by experts to determine the appropriateness of the content and to identify any misunderstandings or omissions [8]. The second version of the questionnaire was reviewed by recognized experts to gather input regarding conceptualization, measurement, analysis, and interpretation of each MovBeh, as well as on the 24-hour movement paradigm.

Fifteen experts from the fields of public health, clinical practice, health promotion and MovBeh epidemiology and psychology were invited to three focus groups. All invited experts agreed to participate. Prior to the focus groups the questionnaire was sent for preliminary consideration. The focus groups were conducted by experienced interviewers [24], following the semi-structured script to understand the experts’ qualitative insights on relevance, comprehensiveness and comprehensibility of the instructions, definitions and examples, items and response options, and item wording and recall period (e.g., What did you think of the questions asked in terms of their relevance?) [8]. Experts were also asked to comment on the domains covered and the logic flow of the questionnaire. The focus groups were conducted using a think-aloud methodology and verbal probing [9, 25], recorded and transcribed for analytic purposes. The questions always followed a qualitative and open-ended format. Afterwards, data were independently analysed by content analysis by two authors (BR, JE) and discussed with the other authors. The insights gained from the focus groups were used to revise the design of the third version of the questionnaire by rewording parts of the text.

### Measurement properties

#### Participants

Participants were eligible if they were aged between 18 and 64.9 years, resided in Portugal, could read and write in Portuguese, non-pregnant or breastfeeding and were generally health (i.e., without a diagnosed disease or condition that would impact their PA). Participants were chosen by a convenience, non-random strategic sampling procedure (i.e., snowball), and 130 participants were invited by direct contact through existing networks. Recruitment took place between May and July of 2023. In addition to the questionnaire, we also collected some demographic information (e.g., age, gender, socio-economic status) and height and weight were collected. Body mass index (BMI) was computed as weight in kilograms divided by height in meters squared.

#### Criterion and convergent validity

A triaxial accelerometer (Actigraph GT3X, Actigraph, Pensacola, Florida, USA), was used to establish the criterion validity of the QMov24h, capturing minute-by-minute observations of whole-body motion. The Actigraph accelerometers have often been used as a concurrent measure to validate MovBeh questionnaires [12, 26]. The Actilife version 6.13.4 software (Actigraph, Pensacola, FL, USA) was used to download and analyse accelerometer data.

Participants were asked to wear the accelerometer on their right hip during 24-hour for 7 days, except for water-based activities. Seven days of accelerometery monitoring has been shown to capture more than 80% of the interindividual variation in adult physical inactivity and activity [27]. To inclusion criterion was applied whereby participants were only included if they had worn the accelerometer for a minimum of 4 days, including one weekend day (≥16 hours per day was considered a valid day). All participants received verbal and written instructions for the proper use of the accelerometer and were instructed to keep the monitor snugly against the body so that it was not allowed to flop around.

Data were collected with a sampling rate of 100 Hz and reintegrated for analysis purposes into 60 seconds epochs. The accelerometer count data were summarized in terms of hours/minutes per day and week. The cut points used to classify accelerometer data, as sedentary time was defined as 0–99 counts per minute (cpm), for LPA as 100–2019 cpm, MPA as 2020–5998 cpm, and for VPA as >5998 cpm [28]. For the criterion validity, sleep was scored manually through visual inspection of accelerometer graphs, alongside sleep diaries. For 24-hour movement guidelines compliance, we used the adults’ Canadian guidelines as an example, as follows: Seven to nine hours of good-quality sleep, limiting SB to 8 hours or less, at least 150 minutes per week of aerobic MVPA and at least two days of muscle-strengthening activities per week. Participants were categorised as having MovBeh levels above or below the Canadian guidelines’ thresholds. Non-wear time was identified as a continuous period of > 60 min with no activity or with information from the logbook. Data were processed according to standard quality assurance procedures [28].

To support and complete accelerometery data analysis, a MovBeh log was also provided to the participants, so that they could record their activity during the seven days, regarding the hours the participant fell asleep and woke up, time and intensity of strength training, moments when the accelerometer was taken out and reason. Potential irregularities, e.g., problems with the monitor or non-wear time, were also documented. This log also served to evaluate convergent validity for the outcomes that were not possible to validate against accelerometery, such as strength and balance activities. The convergent validity is the extent of the agreement with another (non-criterion) measure that should assess the same behaviour parameter based on face and content validity.

Criterion and convergent validity were further assessed by comparing responses from the questionnaire with data from the accelerometers and movement behaviour log. The evaluated outcomes against the accelerometer were: the time spent on a usual weekday (i.e., workday), on a usual weekend day (i.e., non-workday) and the mean of the weekday and weekend day, for nocturnal sleep, naps, SB and LPA, time and frequency of moderate and vigorous intensities for aerobic PA; time and frequency of aerobic MVPA PA (by summing MPA and VPA variables); time spent in total PA (by summing all PA intensity variable). Time spent in strength and balance activities, was evaluated against the log.

### Reliability

Test–retest reliability was evaluated by asking participants to complete the questionnaire seven days after first taking it. This period was thought to be long enough to ensure that participants could not recall the first moment of the questionnaire filling from memory, and short enough to prevent large changes in MovBeh levels [8]. The evaluated scores were the same as for criterion and convergent validity, plus the MovBeh patterns.

### Data analysis

The distribution of the MovBeh data was examined before analysis. Since some data distributions were nonnormal, nonparametric tests were used. Spearman rank correlations were used to assess questionnaire validity scores, comparing the accelerometer and QMov24h data (criterion validity) and the accelerometer and accelerometer log results (convergent validity).

For interpretation of Spearman’s rho values, standard convention was used: negligible (0.01-0.19); weak (0.20-0.29); moderate (0.30-0.39); strong (0.40-0.69) and very strong (≥0.70) [29]. Based on results from similar studies Spearman’s rho ≥ 0.30 was considered adequate [30, 31]. Bland-Altman plots assessed agreement between QMov24h and accelerometer data [32]. In the Bland-Altman plot, proportional bias was verified via regression analysis.

Percentage of agreement was used to assess adherence to the Canadian 24-hour Movement Guidelines. For percentage of agreement, 0-4% represented no agreement, 4-15% represented minimal agreement, 15-35% represented a weak agreement, 35-63% represented a moderate agreement, 64-81% represented a strong agreement, and 82-100% represented an almost perfect agreement [33, 34]. Sensitivity and specificity analysis were also performed.

Two-way mixed effects, single measure, absolute agreement Intraclass Correlation Coefficients (ICC) [35] with a 95% confidence interval (CI) were calculated to assess test-retest reliability scores from the two administrations of the QMov24h. All assumptions for correlation were examined prior to analyses. ICCs coefficient values of <0.50 indicated poor reliability, 0.50 - 0.75 represented moderate reliability, 0.75-0.90 good reliability, and > 0.90 excellent reliability [35]. To check compliance with guidelines regarding reliability, Cohen’s Kappa and percentage of agreement were computed. For Cohen’s Kappa, 0-0.20 represented no reliability, 0.21-0.39 minimal reliability, 0.40-0.59 weak reliability, 0.60- 0.79 moderate reliability, 0.80-0.90 strong reliability, and > 0.90 almost perfect reliability [33]. For percentage of agreement, the classification mentioned above was used.

Bland-Altman plots were also used to assess agreement between the two administrations of the QMov24h.

All analyses were performed using the Statistical Package for the Social Sciences (SPSS), version 28. Statistical significance was set at *p*<0.05.

## Results

### Development of the questionnaire

#### Face validity by end-users

Twenty-five cognitive interviews were conducted (60% female; 46±18,9 years old; 46.2% with full-time job and 26.9% were undergraduate). The interviews began with participants answering the questionnaire individually and in silence; then participants expressed their views, opinions, and doubts following the interview script. Participants took 13.62±2.82 min to answer the questionnaire.

From the interviews, it became clear that the respondents understood the questionnaire as a whole, agreed with the general structure (divided into sections, by MovBeh) and with the questionnaire layout. All participants understood all the concepts and definitions presented in the questionnaire. However, most participants found it difficult to apply the concept of “usual time” to the questions, particularly those whose routines varied from week to week.

Regarding the introduction section of the QMov24h, all participants considered the introduction clear and understood the recall period; however, some participants did not understand that MovBeh were mutually exclusive. There were some minor suggestions regarding the wording of this questionnaire section (e.g., to explicitly state that one can only do one MovBeh at a time; to improve the visual highlighting of the examples provided in the instructions).

Sleep and SB related questions were considered the easiest to answer, compared to PA questions. All participants considered the questions and response format about sleep and SB relevant, comprehensive, and comprehensible. Specifically, in the sleep questions, all participants understood they were not to consider the time they spent lying down without sleeping. The instructions were also found to be adequate. Six participants considered that the example in the SB section, regarding sitting during meals, should be placed at the beginning of the examples because this time of day was harder to consider.

PA questions were the hardest to recall for all participants, mainly for less active ones. Nevertheless, participants considered the questions and response format relevant, comprehensive, and understandable. In this section of the questionnaire, eight participants suggested changing the layout structure of the questions for MVPA. The instructions of this section of the questionnaire were considered adequate by all participants. However, all participants stated that the information regarding physical activity intensities was too descriptive. They suggested simplifying the text and adding images. This suggestion was taken on board and, during the draft of the second version of the questionnaire, an infographic was developed to explain the concepts and examples of PA.

Overall, the cognitive interviews led to some adjustments in the questionnaire, namely in the layout structure and rewording of some items and instructions. Given that 11 participants left some sections of the questionnaire in blank, because they didn’t do some behaviours (e.g. naps), the instructions of the questionnaire were adjusted to ask participants to answer zero (“0”) whenever they did not perform a given behaviour. No additional items were included in the questionnaire, because all the participants believed that the questionnaire adequately covered their MovBeh.

The research team concluded that the changes made to the first version of the questionnaire did not justify new cognitive interviews with end-users, and consequently decided to finish the face validity process.

#### Content validity

During the interviews, all experts agreed that the questionnaire adequately covered the MovBeh that can be done over 24-hour periods. The interviews with experts focused on analysing the various elements of the questionnaire, including the instructions, descriptions of physical activity intensities, and examples for each MovBeh. The introduction section of the questionnaire was validated by all experts, with minor suggestions for rephrasing some sentences. The order of the items was considered to have logic flow. The experts agreed to change the tables format to save space (i.e., instead of the questions about weekend days being placed below weekdays, the two were placed side by side). Also, the format of the response changed to save space (i.e., changed from ___:___ (hours:minutes) to ___h___min). Overall, the definitions, response format, instructions and recall period were considered adequate.

All experts considered the relevance, comprehensiveness and comprehensibility of the sleep and naps questions adequate.

The relevance, comprehensiveness, and comprehensibility of the total SB questions were also considered adequate. However, the experts considered that from a public health surveillance point of view, it would be more relevant that participants reported their SB during leisure and work time, rather than the screen time and passive transport as outlined in the questionnaire’s first draft. This suggestion was accepted.

All the experts valued the PA infographic explain PA types and intensities, considering it as potentially important to reduce reporting bias. The experts considered PA questions to be relevant, comprehensive, and comprehensible. The layout and structure of the questions were considered innovative and a differentiating factor from existing questionnaires. Additionally, the experts recommended including the same pictograms of the infographic as visual aids for defining concepts throughout the questionnaire.

Overall, the feedback received from the experts was very positive, with few substantial changes being suggested. Refinements based on the suggestions provided by experts were made, and the research team considered that there was no need to repeat the face validity phase with the end-users.

#### Description of the questionnaire’s final version

The QMov24h (See Additional files 1 and 2) is a self-administered recall instrument comprising 56 items from 17 questions across 6 main domains. The questionnaire instructs participants starting from sleep behaviours (naps and nocturnal sleep) through SB (total, leisure, and work) to LPA (aerobic and muscle-strengthening activities), MPA (aerobic and muscle-strengthening activities) and VPA (aerobic and muscle-strengthening activities) and balance activities.

In terms of recall and assessment period and response formats for sleep, SB, and LPA domains, the questionnaire asks for how much time, on average, the respondent spends in each behaviour, in a usual weekday (workday) and weekend day (non-workday) (e.g., *In a typical week, on average, how much time do you spend sitting, reclining, or lying down, per day?*). For MPA, VPA and balance activities, the questions and response format considered each weekday and weekend day (e.g., *Estimate the average time, for each day of the week, that you do moderate-intensity aerobic activity.*). This difference between behaviours was made because sleep, SB and LPA are performed daily. At the same time, MPA and VPA and balance activities tend to be more structured activities that follow weekly patterns.

The outcomes of the QMov24h are the time (hours or minutes) spent per day in sleep and LPA; and the frequency (i.e. number of days); and the time (hours or minutes) spent in MPA (aerobic and strength activities), VPA (aerobic and strength activities) and balance activities, with these being able to be averaged by day. Given the continuous nature of the questionnaire outcomes, all outcomes can be categorized to assess compliance with 24-hour movement guidelines. The questionnaire was structured into three distinct sections: sleep, SB, and PA. These sections can be analysed independently or jointly. An excel sheet to calculate the outcome variables of the QMov24h is presented in Additional file 3.

Recognizing the complexity of responding to questionnaires measuring complex behaviours, it was acknowledged that incorporating relevant cues and examples of the behaviours of interest was crucial for designing effective assessment tools [36]. For instance, to facilitate participants’ understanding of the applicable activities for each question, the questionnaire provided a definition and multiple examples for each behaviour and intensity.

Additional file 2 contains a copy of the questionnaire translated into English. The translation was checked by two independent researchers fluent in both Portuguese and English through back translation. Any discrepancies were resolved through agreement.

### Measurement properties

#### Socio-demographic characteristics

A total of 130 adults were invited for the criterion and convergent validity and reliability studies, of these, 100 participants completed at least the first questionnaire and provided sufficient valid days of accelerometery (criterion validity study), and 117 completed the questionnaire on both occasions (convergent validity and reliability studies). Participants of the face-validity study did not participate in the criterion and convergent validity and reliability studies. For the criterion validity study (*n*=100; 61% women) participants were 31.33 ± 13.68 years old, 33% worked full-time, and 44% were university students. For the convergent validity and reliability studies (*n*=117; 58.1% women) participants were 30.95 ± 13.56 years old, 34% worked full-time, and 54% were university students (Table 1). Participants took between 10 to 15 min to respond the questionnaire. On average the answer to all questions summed 23 hours and 13.2 min.

**Table 1:** Participants’ characteristics

	**Validity (*n*=100)**	**Reliability (*n*=117)**
**Gender**		
Men	39 (39.0%)	49 (41.9%)
Women	61 (61.0%)	68 (58.1%)
**Age (years)**	31.3 ± 13.7	31.0 ± 13.6
**Education**		
Less than high school	8 (8%)	8 (6.8%)
High school/Vocational education	27 (27%)	36 (30.8)
University degree incomplete	19 (19%)	20 (17.1)
Undergraduate	30 (30%)	33 (28.2%)
Master’s degree	14 (14%)	17 (14.5)
PhD	2 (2%)	3 (2.6%)
**Employment**		
Full-time	34 (34%)	39 (33.3%)
Part-time	4 (4%)	4 (3.4%)
Unemployed	2 (2%)	2 (1.7%)
Student	44 (44%)	53 (54.3%)
Retired	5 (5%)	5 (4.3%)
Domestic work	10 (10%)	13 (11.1%)
Inability to work	1 (1%)	1 (0.9%)
**Body mass index (kg/m**^**2**^**)**	23.52 ± 3.67	23.38 ± 3.53

Data are n (%) or mean±SD

### Validity results

#### Criterion-validity

**Fig. 1:**
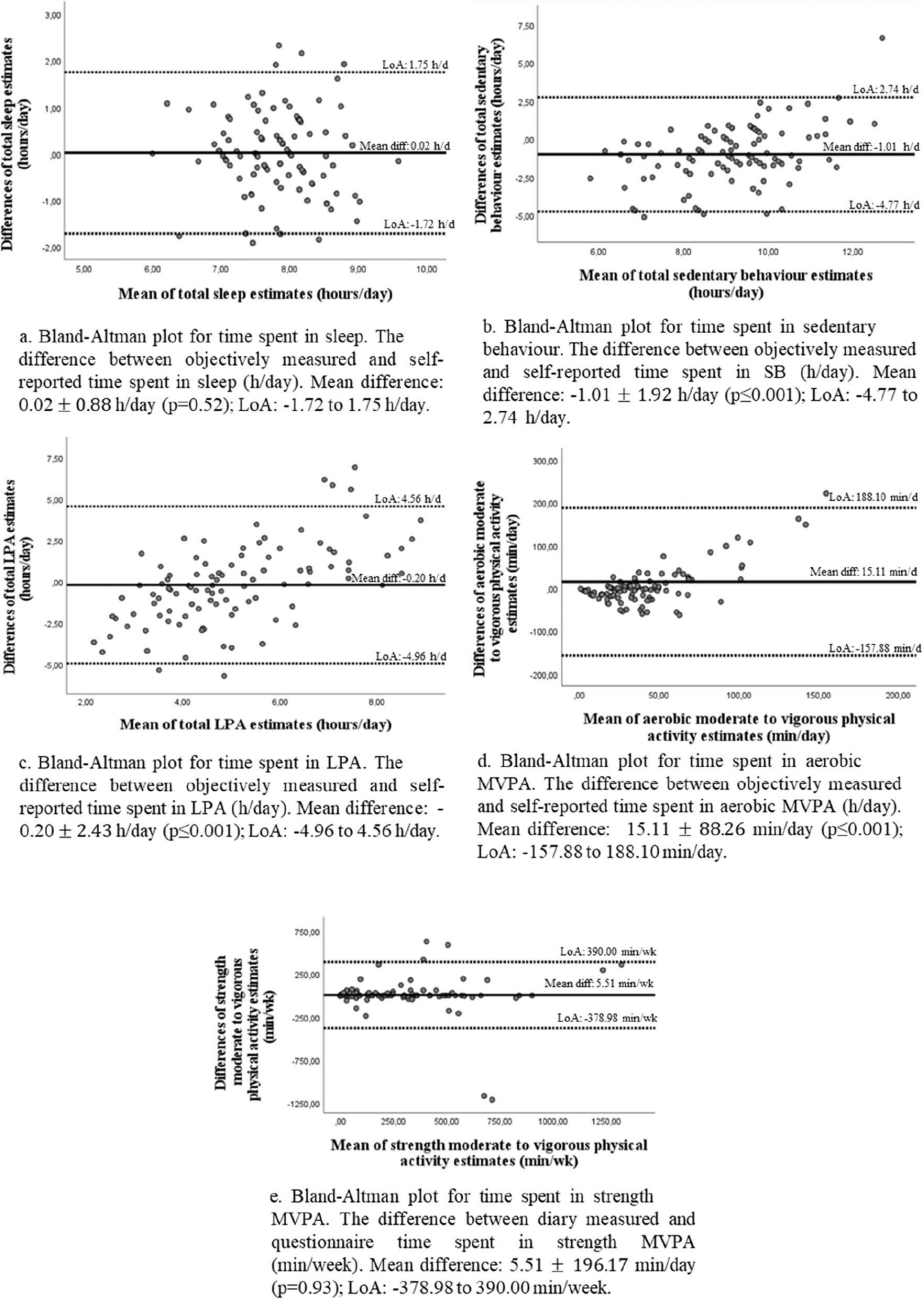
Bland Altman plots for validity (QMov24h against accelerometer or diary). Legend: LPA: Light Physical Activity; MPA; Moderate Physical Activity; VPA: Vigorous Physical Activity; MVPA: Moderate to Vigorous Physical Activity; h: Hours; d: Day; min: Minutes; wk: Week

As presented in Table 2, all main variables showed adequate criterion validity (i.e., rho ≥ 0.30; *p*<0.05). Regarding nocturnal sleep variables, Spearman correlations showed weak to strong associations between the accelerometer and sleep variables as measured by the questionnaire (rho from 0.27 to 0.41; *p*<0.05). Figure 1a. illustrates the Bland Altman plot for total sleep showing good agreement between the accelerometer and QMov24h, without proportional bias (*p*=0.52), with a mean difference of 0.02 ± 0.88 h/day and a Limits of Agreement (LoA) between -1.72 and 1.75 h/day. For SB variables, Spearman correlations showed moderate to strong associations between the accelerometer and all the SB variables (rho from 0.36 to 0.43; *p*<0.001). Figure 1b. illustrates the Bland-Altman plot for total SB, showing good agreement between the accelerometer and QMov24h, with a slight significant underestimation of SB from the questionnaire (*p*≤0.001), with a mean difference of -1.01 ± 1.92 h/day and LoA between -4.77 and 2.74 h/day. For LPA variables, Spearman correlations showed weak to moderate associations between the accelerometer and LPA variables as measured by questionnaire (rho from 0.25 and 0.33; *p*<0.001). Figure 1c. illustrates the Bland-Altman plot for total LPA, showing good agreement between the accelerometer and QMov24h, with a slight underestimation of LPA from the questionnaire (*p*≤0.001), with a mean difference of -0.20 ± 2.43 h/day and a LoA between -4.96 and 4.56 h/day). For aerobic MPA, VPA, MVPA, and total PA variables (min/day), Spearman correlations showed moderate to strong associations between the accelerometer and all these PA variables (rho from 0.37 to 0.57; *p*<0.001). Figure 1d. illustrates the Bland-Altman plot for aerobic MVPA, showing a good agreement between the accelerometer and QMov24h, with a significant overestimation of aerobic MVPA from the questionnaire (*p*≤0.001), with a mean difference of 15.11 ± 88.26 min/day but wide LoA between -157.88 and 188.10 min/day). As supplementary material the Bland-Altman plot with MovBeh expressed as proportions of 24-hour are presented (Additional file 4).

**Table 2:** Validity of movement behaviours variables from the QMov24h against accelerometer (*n*=100)

**Movement behaviour variable**	**Questionnaire variable description**	**Questionnaire data**	**Accelerometer data**	**rho**	**p**
		**Mean minutes/hours (95% CI)**	**Mean minutes/hours (95% CI)**		
**Nocturnal sleep**					
**Weekday/workday (h/day)**	Hours of sleep at night on a weekday/workday	7.30 (7.13 – 7.47)	7.69 (7.51 – 7.86)	0.410	<0.001
**Weekend day/non-workday (h/day)**	Hours of sleep at night on a weekend day/non-workday	8.36 (8.17 – 8.55)	8.18 (7.90 – 8.47)	0.272	0.006
**Total sleep (h/day)**	Mean hours of sleep at night on a weekday/workday and on a weekend day/non-workday	7.83 (7.68 – 7.98)	7.81 (7.65 – 7.97)	0.343	<0.001
**Sedentary behaviour**					
**Weekday/workday (h/day)**	Hours of SB on a weekday/workday	8.81 (8.42 – 9.21)	9.81 (9.54 – 10.07)	0.357	<0.001
**Weekend day/non-workday (h/day)**	Hours of SB on a weekend day/non-workday	8.35 (7.76 – 8.93)	9.39 (9.02 – 9.77)	0.404	<0.001
**Total SB (h/day)**	Mean hours of SB on a weekday/workday and on a weekend day/non-workday	8.59 (8.18 – 9.00)	9.61 (9.34 – 9.87)	0.428	<0.001
**Light PA**					
**Week/workday (h/day)**	Hours of aerobic light PA + strength light PA on a weekday/workday	5.40 (4.79 – 6.02)	5.10 (4.83 – 5.37)	0.331	<0.001
**Weekend/non-workday (h/day)**	Hours of aerobic light PA + strength light PA on a weekend day/non-workday	4.48 (3.91 – 5.04)	5.10 (4.77 – 5.43)	0.248	0.013
**Total Light PA (h/day)**	Mean hours of aerobic light PA + strength light PA on a weekday/workday and on a weekend day/non-workday	4.95 (4.45 – 5.45)	5.15 (4.89 – 5.41)	0.310	0.002
**Aerobic Moderate and Vigorous PA**					
**Aerobic moderate PA (min/day)**	Average min per day of aerobic moderate PA in minutes	39.25 (27.39 – 51.11)	39.45 (34.43 – 42.46)	0.500	<0.001
**Aerobic vigorous PA (min/day)**	Average min per day of aerobic vigorous PA in minutes	16.52 (8.52 – 24.52)	2.24 (1.39 – 3.09)	0.570	<0.001
**Aerobic MVPA (min/day)**	Sum of calculated aerobic moderate PA and vigorous PA mins per day	55.77 (36.60 – 74.94)	40.67 (36.23 – 45.10)	0.534	<0.001
**Total PA**					
**Total aerobic PA (min/day)**	Sum of average min per day of light PA and aerobic MVPA	353.02 (314.34 – 391.70)	349.82 (333.51 – 366.12)	0.372	<0.001
**Total aerobic and strength PA (min/day)**	Sum of average min per day of light PA and aerobic and strength PA	387.26 (345.44 – 429.09)	383.95 (365.18 – 402.71)^a^	0.429	<0.001

*SD* Standard Deviation, *CI* Confidence Interval, *rho* Spearman Correlation, *p p*-value, *PA* Physical Activity, *MVPA* Moderate to Vigorous Physical Activity

^a^Strength PA was compared against diary

Regarding compliance with the 24-hour movement guidelines, when variables were categorized as below or above the guidelines’ thresholds (e.g. 150 min/week of MVPA), the percentages of agreement between the questionnaire responses and the accelerometer results showed a strong agreement for sleep compliance (69%) and an almost perfect agreement for SB (76%), aerobic MVPA (81.2%) and strength MVPA (94.3%) compliance. Sensitivity and specificity were stated in Table 5.

#### Convergent validity

Convergent validity analyses are presented in Table 3. All main variables showed adequate convergent validity (i.e., rho ≥ 0.30; *p*<0.05), when compared against the MovBeh diaries. For naps variables, Spearman correlations showed weak to moderate associations between the MovBeh diary and naps variables assessed by questionnaire (rho from 0.23 to 0.37; *p*<0.05). For strength MPA, VPA, MVPA variables (both hours/week and times/week), Spearman correlations showed very strong associations between the MovBeh diary and all these PA variables as assessed by questionnaire (rho from 0.85 to 0.97; *p*<0.001). Figure 1e. illustrates the Bland-Altman plot for strength MVPA, showing a good agreement between the diary and QMov24h, without proportional bias from the questionnaire (*p*=0.93), with a mean difference of 5.51 ± 196.17 min/day and a LoA between -390.00 and 379.00 min/day. For balance activities variables (hours/week and times/week), Spearman correlations showed very strong associations between the diary and all balance variables (rho from 0.73 to 0.80; *p*<0.001).

**Table 3:** Validity of movement behaviours from the QMov24h variables against diary (*n*=117)

**Movement behaviour variable**	**Questionnaire variable description**	**Questionnaire data**	**Diary data**	**rho**	**p**
		**Mean minutes/hours (95% CI)**	**Mean minutes/hours (95% CI)**		
**Naps**					
**Week/workday (h/day)**	Hours of naps on a weekday/workday	0.18 (0.09 – 0.27)	0.01 (0.00 – 0.02)	0.367	<0.001
**Weekend/non-workday (h/day)**	Hours of naps on a weekend day/non-workday	0.22 (0.10 – 0.34)	0.27 (-0.24 – 0.77)	0.232	0.012
**Both (h/day)**	Mean hours of naps on a weekday/workday and on a weekend day/non-workday	0.20 (0.10 – 0.30)	0.01 (0.00 – 0.02)	0.355	<0.001
**Strength moderate PA (min/week)**	Sum of all min per day of strength moderate PA	137.12 (104.66 – 169.70)	140.51 (102.15 – 178. 87)	0.850	<0.001
**Strength moderate PA (times/week)**	Sum of the number of days with moderate strength exercise reported	2.71 (2.21 – 3.21)	2.57 (2.09 – 3.05)	0.889	<0.001
**Strength vigorous PA (min/week)**	Sum of all min per day of strength vigorous PA	95.73 (65.88 – 125.57)	110.76 (83.11 – 138.43)	0.966	<0.001
**Strength vigorous PA (times/week)**	Sum of the number of days with vigorous strength exercise reported	1.92 (1.46 – 2.38)	1.85 (1.39 – 2.31)	0.910	<0.001
**Strength MVPA (min/week)**	Sum of calculated strength moderate PA and vigorous PA minutes per week	241.75 (189.88 – 293.62)	236.24 (184.08 – 288.40)	0.878	<0.001
**Strength MVPA (times/week)**	Sum of the number of days with moderate or vigorous strength exercise reported	3.35 (2.85 – 3.85)	3.13 (2.65 – 3.61)	0.899	<0.001
**Balance activity (min/week)**	Sum of all min per day of balance activities	64.31 (29.36 – 99.27)	41.34 (20.01 – 62.66)	0.795	<0.001
**Balance activity (times/week)**	Sum of the number of days with balance activities reported	1.22 (0.81 – 1.63)	0.79 (0.47 – 1.12)	0.731	<0.001

*SD* Standard Deviation, *CI* Confidence Interval, *rho* Spearman Correlation, *p p*-value, *PA* Physical Activity, *MVPA* Moderate to Vigorous Physical Activity

#### Reliability

Table 4 presents the results of test-retest analyses comparing the first administration of the QMov24h with the second.

**Table 4:** Test-retest reliability (7 days apart) of movement behaviours variables from the QMov24h (*n*=117)

**Movement behaviour variable**	**T1**	**T2**	**ICC (95% CI)**	**p**
	**Mean minutes/hours/months (95% CI)**	**Mean minutes/hours/months (95% CI)**		
**Nocturnal sleep**				
Week/workday (h/day)	7.26 (7.11 – 7.42)	7.21 (7.05 – 7.37)	**0.788 (0.71 – 0.85)**	<0.001
Weekend/non-workday (h/day)	8.36 (8.19 – 8.53)	8.36 (8.19 – 8.53)	**0.775 (0.69 – 0.84)**	<0.001
Both (h/day)	7.81 (7.68 – 7.95)	7.78 (7.65 – 7.91)	**0.776 (0.69 – 0.84)**	<0.001
**Naps**				
Week/workday (h/day)	0.18 (0.09 – 0.27)	0.21 (0.10 – 0.31)	**0.643 (0.52 -0.74)**	<0.001
Weekend/non-workday (h/day)	0.22 (0.10 – 0.34)	0.24 (0.13 – 0.36)	**0.710 (0.61 -0.79)**	<0.001
Both (h/day)	0.20 (0.10 – 0.30)	0.20 (0.10 – 0.30)	**0.750 (0.66 – 0.82)**	<0.001
Patterns of sleep (Months; *n*=81)	49.53 (34.94 – 64.13)	43.35 (30.17 – 56.52)	**0.896 (0.84 – 0.93)**	<0.001
**Sedentary behaviour**				
Week/workday (h/day)	8.62 (8.22 – 9.02)	8.39 (7.97 – 8.82)	**0.875 (0.82 – 0.91)**	<0.001
Weekend/non-workday (h/day)	8.04 (7.44 – 8.64)	7.63 (7.12 – 8.15)	**0.748 (0.65 – 0.82)**	<0.001
Both (h/day)	8.34 (7.91 – 8.77)	7.99 (7.58 – 8.39)	**0.797 (0.71 – 0.86)**	<0.001
Sedentary behaviour on work	5.07 (4.61 – 5.54)	5.17 (4.73 – 5.60)	**0.927 (0.90 – 0.95)**	<0.001
Sedentary behaviour on leisure	3.25 (2.81 – 3.69)	3.07 (2.61 – 3.53)	**0.811 (0.74 – 0.87)**	<0.001
Patterns of sedentary behaviour (Months; *n*=66)	38.00 (23.42 – 54.58)	38.63 (24.03 – 53.24)	**0.962 (0.94 – 0.98)**	<0.001
**Light PA**				
Week/workday (h/day)	5.24 (4.68 – 5.80)	5.19 (4.67 – 5.71)	**0.844 (0.78 – 0.89)**	<0.001
Weekend/non-workday (h/day)	4.50 (3.96 – 5.03)	4.35 (3.84 – 4.87)	**0.833 (0.77- 0.88)**	<0.001
Both (h/day)	4.88 (4.41 – 5.35)	4.77 (4.31 – 5.23)	**0.840 (0.78 – 0.87)**	<0.001
Patterns of light PA (Months; *n*=71)	35.64 (22.28 – 49.01)	30.60 (18.83 – 42.37)	**0.869 (0.80 – 0.91)**	<0.001
Aerobic moderate PA (min/day)	40.17 (29.58 – 50.76)	40.59 (31.08 – 50.10)	**0.827 (0.76 – 0.88)**	<0.001
Strength moderate PA (min/week)	137.18 (104.66 – 169.70)	149.87 (110.94 – 188.80)	**0.726 (0.63 – 0.80)**	<0.001
Strength moderate PA (times/week)	2.71 (2.21 – 3.21)	2.62 (2.12 – 3.13)	**0.891 (0.85 – 0.92)**	<0.001
Aerobic vigorous PA (min/day)	17.80 (10.70 – 24.91)	12.31 (8.18 – 16.45)	**0.717 (0.61 – 0.80**	<0.001
Strength vigorous PA (min/week)	95.73 (65.88 – 125.57)	72.10 (48.01 – 96.20)	**0.812 (0.73 – 0.87)**	<0.001
Strength vigorous PA (times/week)	1.92 (1.46 – 2.38)	1.73 (1.29 – 2.17)	**0.824 (0.76 – 0.88)**	<0.001
Aerobic MVPA (min/day)	55.97 (41.01 – 74.93)	52.91 (40.60 -65.21)	**0.820 (0.75 – 0.87)**	<0.001
Strength MVPA (min/week)	241.75 (189.88 – 293.62)	221.37 (175.45 – 268.49)	**0.748 (0.66 – 0.82)**	<0.001
Strength MVPA (times/week)	3.35 (2.85 – 3.85)	3.14 (2.64 – 3.63)	**0.835 (0.77 – 0.88)**	<0.001
Aerobic and strength MVPA (min/day)^a^	92.51 (70.63 – 114.39)	84.68 (68.58 -100.65)	**0.801 (0.73 – 0.86)**	<0.001
Total aerobic PA (min/day)	350.75 (315.75 – 385.75)	339.18 (308.23 – 370.13)	**0.828 (0.76 – 0.88)**	<0.001
Total aerobic and strength PA (min/day)^a^	385.29 (347.45 – 423.12)	370,89 (338.36 – 403.42)	**0.820 (0.75 – 0.87)**	<0.001
Patterns of MVPA (Months; *n*=69)	34.64 (20.26 – 49.03)	30.53 (16.82 – 44.23)	**0.895 (0.84 – 0.93)**	<0.001
Balance activity (min/week)	63.76 (29.09 – 98.43)	45.60 (26.00 – 65.19)	**0.380 (0.21 – 0.52)**	<0.001
Balance activity (times/week)	1.22 (0.81 – 1.63)	1.12 (0.72 – 1.52)	**0.730 (0.63 – 0.80)**	<0.001
Patterns of balance activity (Months; *n*=51)	34.64 (20.53 – 49.03)	30.53 (16.82 – 44.23)	**0.895 (0.84 – 0.93)**	<0.001

*SD* Standard Deviation, *CI* Confidence Interval, *ICC* Intraclass correlation, *p p*-value, *PA* Physical Activity, *MVPA* Moderate to Vigorous Physical Activity

^a^Mean of minutes per day of aerobic MVPA + (Mean of minutes per week of strength MVPA / 7)

**Fig. 2:**
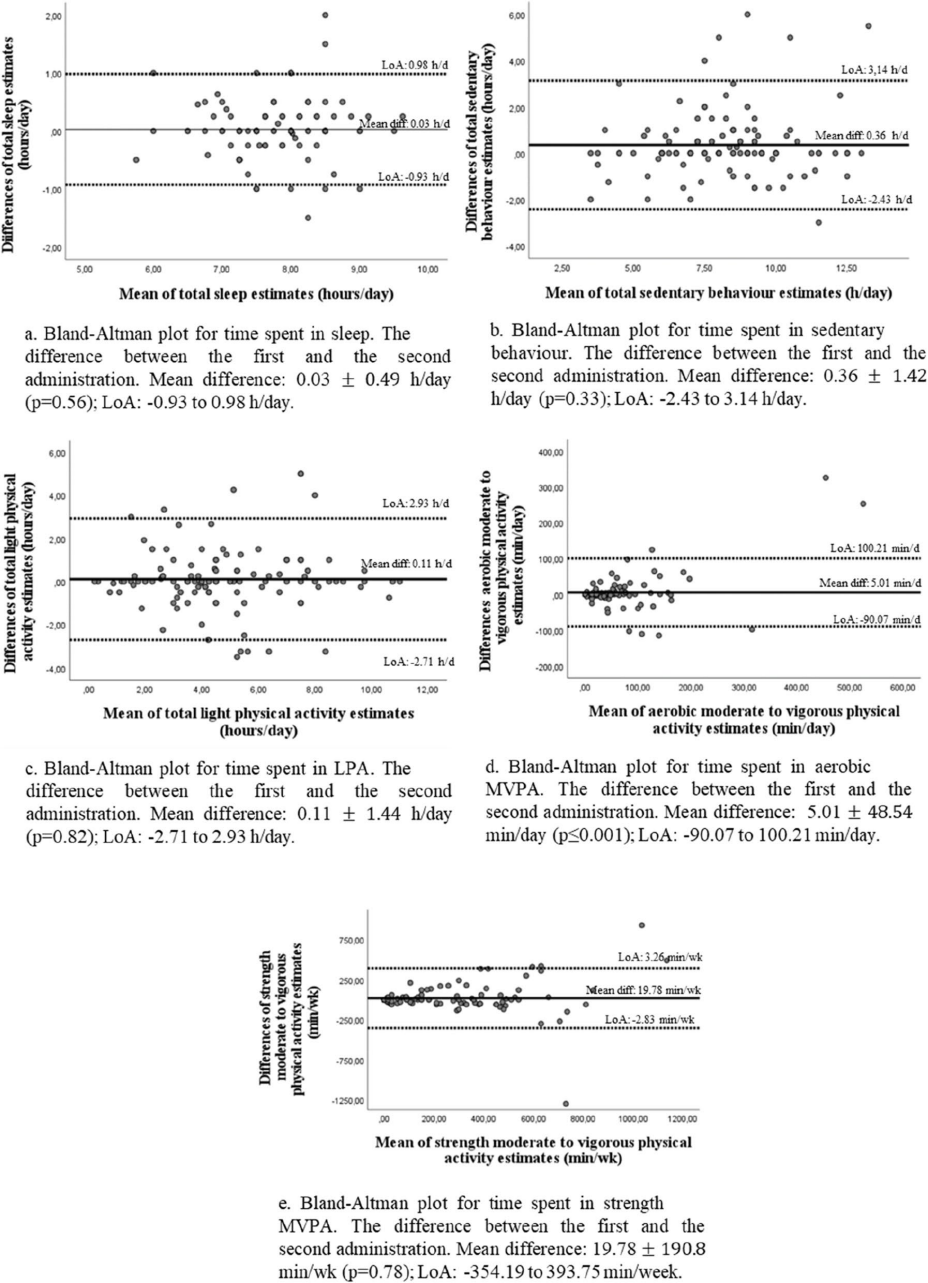
Bland Altman plots for reliability (first against second administration of from the QMov24h). Legend: LPA: Light Physical Activity; MPA; Moderate Physical Activity; VPA: Vigorous Physical Activity; MVPA: Moderate to Vigorous Physical Activity; h: Hours; d: Day; min: Minutes; wk: Week

For nocturnal sleep variables, ICCs showed good agreement between the first and second administration of the questionnaire (ICC from 0.78 to 0.79; *p*<0.001). Figure 2a. illustrates the Bland-Altman plot for total sleep, showing good reliability without proportional bias (*p*=0.56) between the two administrations of the questionnaire, with a mean difference of 0.03 ± 0.49 h/day and a LoA between -0.93 and 0.98 h/day. For naps variables, ICCs showed significant and moderate to good agreements between the first and second administrations (ICC of 0.64 to 0.75; *p*<0.001). For SB variables, ICCs showed good to excellent agreements between the first and second administration of the questionnaire (ICC from 0.75 to 0.93; *p*<0.001). Figure 2b. illustrates the Bland-Altman plot for total SB, showing good reliability without proportional bias (*p*=0.33) between the two administrations of the questionnaire, with a mean difference of 0.36 ± 1.42 h/day; LoA between -2.43 and 3.14 h/day. For LPA variables, ICCs showed good agreement between the first and second administration of the questionnaire (ICC from 0.83 to 0.84; *p*<0.001). Figure 2c. illustrates the Bland-Altman plot for total LPA, which showed good reliability without proportional bias (*p*=0.82) between the two administrations of the questionnaire, with a mean difference of 0.11 ± 1.44 h/day; LoA between -2.71 and 2.93 h/day. For aerobic MPA, VPA, MVPA, total PA, ICCs showed moderate to good agreements between the first and second administration of the questionnaire (ICC from 0.72 to 0.83; *p*<0.001). Figure 2d illustrates the Bland-Altman plot for aerobic MVPA, indicating good reliability with a slight overestimation (*p*≤0.001) of the first administration, with a mean difference of 5.01 ± 48.54 min/day; LoA between -90.07 and 100.21 min/day. For strength MPA, VPA, MVPA variables (hours/week and times/week), ICCs showed significant and moderate to good agreements between the first and second administration of the questionnaire (ICC from 0.73 to 0.89; *p*<0.001). Figure 2e illustrates the Bland-Altman plot for strength MVPA, indicating good reliability without proportional bias (*p*=0.78) between the two administrations of the questionnaire, with a mean difference of 19.78 ± 190.80 min/day; LoA between -393.75 and -354.19 min/day). For aerobic and strength MVPA and total PA, ICCs showed good agreement between the first and second administration of the questionnaire (ICC of 0.80 and 0.82, respectively; *p*<0.001). For balance activities variables (hours/week and times/week), ICCs showed poor to moderate agreement between the first and second administration of the questionnaire (ICC of 0.38 and 0.73, respectively; *p*<0.001).

Regarding compliance with the 24-hour movement guidelines, when variables were categorized as below or above the guidelines’ thresholds (e.g. 150 min/week of MVPA), the percentages of agreement between the two administrations of the questionnaire results showed minimal to strong reliability (k from 0.38 to 0.87; *p*<0.001), while the percentages of agreement represented a strong agreement for sleep compliance (80.3%) and an almost perfect agreement for SB (85.3%), aerobic MVPA (94.0%) and strength MVPA (88.0%) compliance. Sensitivity and specificity were stated in Table 5.

**Table 5:** Percentage of agreement between the compliance with the Canadian 24h movement guidelines and the from the QMov24h data (dichotomized)

	**Validity**^a^			**Reliability**^b^		
	**% of agreement**	**Sensitivity**	**Specificity**	**Kappa (*p*-value; % of agreement)**	**Sensitivity**	**Specificity**
Compliance with sleep guidelines	69%	76.3%	40.0%	0.38 (*p*=<0.001; 80.3%)	81.4%	73.3%
Compliance with SB guidelines	75.8%	90.0%	74.2%	0.70 (*p*=<0.001; 85.3%)	74.5%	95.1%
Compliance with aerobic MVPA guidelines	81.2%	80.8%	82.6%	0.87 (*p*=<0.001; 94,0%)	96.1%	90.2%
Compliance with strength MVPA guidelines	94.3%	97.3%	88.4%	0.74 (*p*=<0.001; 88.0%)	93.2%	79.5%

^a^Comparing questionnaire with accelerometer

^b^Comparing first questionnaire with second questionnaire

## Discussion

The *24-hour Movement Questionnaire* (QMov24h) was developed to assess population MovBeh levels and compliance with the 24-hour movement guidelines. The QMov24h presented adequate content and face validity. The QMov24h showed moderate criterion validity for sleep, LPA and total aerobic PA, and strong criterion validity for SB and aerobic MVPA. The questionnaire also displayed good agreement with accelerometer measures in all main MovBeh variables, with SB and LPA being slightly underestimated in the questionnaire, as well as with aerobic MVPA being marginally overestimated by the questionnaire. The QMov24h presented moderate convergent validity for naps and very strong convergent validity for all strength and balance variables. Reliability of the QMov24h varied from good to excellent in all questionnaire variables; between the first and second administration of the questionnaire, there was good agreement for all questionnaire variables, with aerobic MVPA being slightly overestimated on the first administration of the questionnaire. Regarding compliance with the 24-hour movement guidelines, the questionnaire revealed strong to almost perfect percentage of agreement (criterion validity) with accelerometery and minimal to strong reliability between the first and second administrations of the questionnaire.

On average the answer to all questions of the QMov24h summed 23 hours and 13.2 min. This is an important result. Because, from a global/daily point of view, the closer the sum of the questionnaire is to 24 hours, the lower the risk of overestimation or underestimation the questionnaire has.

The QMov24h showed adequate face and content validity following minor adjustments to wording and layout structure, which were based on feedback from end-users and experts during the questionnaire's development process. These forms of validity are crucial for questionnaire development, but are often neglected [24]. In fact, only a few studies have assessed the relevance, comprehensiveness, and comprehensibility of questionnaires [8].

### Validity and reliability of sleep variables

The questionnaire presented adequate validity and reliability for sleep variables, consistent with the validation results of other sleep questionnaires. However, caution should be exercised when making direct comparisons, as other validations of sleep questionnaires have typically focused on the validity of summary scores based on multiple dimensions of sleep, including sleep quality, rather than specifically on sleep duration or against different criterion methods [37, 38]. For example, the validation study of the Daily Activity Behaviours Questionnaire (DABQ) [39], showed greater criterion validity than our QMov24h; however, it was conducted with ActivPaL inclinometers placed on the thigh. Nevertheless, the QMov24h showed better reliability than the DABQ. Other sleep questionnaires tend to overestimate sleep duration when compared with accelerometry [38, 40], and the QMov24h showed a good agreement without proportional bias between the two methods.

The DABQ [39] did not validate the questions regarding naps, which is consistent with a recent systematic review that reported the lack of studies validating questions for naps [12]. We addressed this gap, and the QMov24h showed adequate convergent validity for this sleep variable.

### Validity and reliability of sedentary behaviour variables

All SB variables showed adequate criterion validity and good to excellent reliability. Despite having different methodologies (e.g., accelerometer model, body placement, cut-points, etc.), the total SB variable from the QMov24h (rho=0.43) achieved slightly better validity results when compared with most of the SB questionnaires (*r*=0.32) [41]. Additionally, the QMov24h had better SB validity results than well-established questionnaires, such as the usual week IPAQ long (rho from 0.14 to 0.22) and short (rho from 0.07 to 0.26, against accelerometers) forms [31], and the Global Physical Activity Questionnaire (rho=-0.20, against pedometers) [16]. QMov24h showed a good agreement with a slight underestimation of the questionnaire (-1.01 ± 1.92 h/day), which is similar to some studies [39, 42], but better than others [43–45]. The positive proportional bias showed that the questionnaire tends to underestimate the SB of those who spend more time in sedentary activities and overestimate the values of those who spend less time in these activities. Regarding the wide limits we observed a small deviation at the individual level, which shows that this questionnaire is better measuring SB at group level. However, wider limits are common in SB questionnaires [42, 46], probably due to some limitations of the objective measures and/or the difficulty reporting SB.

In terms of reliability, the QMov24h's total SB variable (ICC=0.80) outperformed most SB questionnaires (ICC=0.66) [41]. Furthermore, the QMov24h demonstrated similar reliability to the long (rho = 0.74 to 0.93) and short (rho = 0.58 to 0.94) forms of the usual week IPAQ [31]. QMov24h exhibited good agreement in test-retest reliability, without proportional bias between the two questionnaire administrations. In contrast, some studies reported a slight overestimation in the results of the initial questionnaire administration [47].

Regarding the reliability of SB at work (ICC=0.93) and during leisure time (ICC=0.81), the QMov24h yielded better results than the Sedentary, Transportation and Activity Questionnaire (STAQ) [45] and the SIT-Q [47], two questionnaires devoted to the duration of SB, with ICCs of 0.71 and 0.67, and ICC of 0.81 and 0.61, respectively. It is worth pointing out that differentiating between behavioural domains through accelerometry alone can be difficult, so testing their criterion validity is a complex challenge, although it has been done in studies using video cameras that record contextual information about behaviours to help define each specific domain [48].

### Validity and reliability of PA variables

Possibly because LPA has only recently been included in the WHO guidelines [15], and given that evidence on the health-related benefits of LPA is quite recent [49, 50], there are few questionnaires assessing it [12, 51, 52]. For example, the widely used IPAQ [14] and Global Physical Activity Questionnaire (GPAQ) [16] do not assess LPA. The Community Health Activities Model Program for Seniors questionnaire has been modified to measure LPA; however, with low validity (*r*=0.06) and only in older adults [53]. The Sedentary Behaviour and Light-intensity Physical Activity Questionnaire was recently developed to measure LPA and SB, and has shown adequate validity (rho=0.43) and reliability (ICC=0.70) [54]. However, it has limitations as it does not measure all PA intensities or sleep, which is important for measuring the 24-hour movement behaviour composition. Additionally, it was developed with a clinical population, so its measurement properties in the general population are unknown. The DABQ [39] showed an adequate validity (rho=0.45) for LPA; although, unlike QMov24h, this questionnaire does not include specific questions for LPA, rather, LPA is *a posteriori* calculated as the remaining time to the total time spent in MVPA. On the other side, QMov24h displayed greater reliability than the DABQ for total LPA (ICC=0.69 vs. ICC=0.84). QMov24h showed a higher agreement for LPA (mean difference=0.20 h/day, i.e., 12 min/day) with accelerometery in the Bland Altman analysis than DABQ (mean difference=134.5 min/day). Nevertheless, the overestimation is contingent upon the level of the LPA and the wide limits demonstrate that this questionnaire is more precise at the group level than at the individual level. As such, QMov24h is one of the first valid and reliable questionnaires focused on assessing all MovBeh, including LPA. As movement behaviours are difficult to self-report in detail, we decided to construct a questionnaire that would give respondents a clear and as concise as possible detailed information on all movement behaviours. We also stated at the beginning of the questionnaire that in a 24-hour day, each movement behaviour can only be done one at a time. Particularly for LPA, (as it is an unstructured and incidental activity), the detailed information provided supplemented with pictograms, requesting data on light aerobic and strength activities, were strategies that may have contributed to the validity of the LPA questions.

The QMov24h study found significant moderate to strong significant associations between accelerometery and aerobic MPA, VPA and MVPA, and total aerobic PA variables (in min/day) with Spearman correlation coefficients ranging from 0.37 to 0.57 (*p*<0.001). These results are comparable to or even better than those reported in other validation studies, such as the IPAQ and GPAQ, which reported Spearman correlation coefficients between 0.27 and 0.49 [30, 31, 55–57]. For example, a study validating the GPAQ for the European context showed negligible validity for aerobic MPA (rho=0.16) [58], whereas the QMov24h exhibited strong validity (rho=0.50). Also, with a Spearman correlation of 0.53 for aerobic MVPA, the QMov24h seems to have a better criterion validity for this intensity than almost all of the questionnaires included in a recent systematic review on the measurement properties of MovBeh questionnaires [12]. Furthermore, in the present study, Bland Altman analysis presented a good agreement between the accelerometer and QMov24h, albeit with an overestimation of aerobic MVPA from the questionnaire of 15.11±88.26 min/day, with a positive proportional bias. The positive proportional bias showed that the questionnaire tends to overestimate the MVPA of those who do little MVPA and underestimate the values of those who perform more MVPA. In comparison, other questionnaires overestimated MVPA by 46 min/day (GPAQ) [59] and 76 min/day (IPAQ) [59], against accelerometery. As with other questionnaires [59–62], there were wide LOAs for MVPA. This wide LoAs show that we should be cautious when using this questionnaire to assess MVPA at the individual level. However, regardless of these higher LoA, most observations fell within a low range of values, with the differences between methods being higher, only for a few participants that presented much higher levels of MVPA (values higher than 80 min/day), which are rare at a population level. Thus, the Bland-Altman analysis showed reasonable agreement between methods for MVPA, but one must be aware that for higher levels of MVPA (i.e., much higher the PA recommendations), the questionnaire may be less suitable to assess this specific intensity. Regarding test-retest reliability, the QMov24h showed moderate to good agreement (ICC of 0.72 to 0.83; *p*<0.001). These results align with those from GPAQ and are superior to the IPAQ [51]. Compared with DABQ [39], a questionnaire that also considers the 24h MovBeh paradigm, QMov24h displayed better criterion validity (rho: 0.38 vs 0.53) and reliability (ICC: 0.65 vs. 0.82) for aerobic MVPA variables. In the face validity focus groups, it was reported that with a response format identical to the IPAQ (average per day of hours and minutes per PA intensity) was harder to report for MVPA. Therefore, for MVPA, our strategy was to develop a response format where respondents were asked to report their MVPA on a daily basis, making it easier to remember what they did each day of the week.

The strength MPA, VPA, and MVPA variables (hours/week and times/week) showed very strong associations with the diary (rho from 0.85 to 0.97; *p*<0.001). Having a questionnaire that includes items related to muscle strengthening activities is of utmost importance because, although muscle strengthening activities have been included in the guidelines since 1995 [63], it is not a PA type that is usually measured in clinical and surveillance studies. Potentially because until now, there were no properly validated questionnaires that included questions/items on muscle strengthening activities [12]. Although the current WHO PA guidelines [15], and the Canadian 24-hour Movement guidelines [4] only mention the frequency of muscle-strengthening activities (twice a week), our questionnaire goes beyond that and also records the time spent on these activities. In this regard it would be ideal to have a questionnaire that included other domains of strength, such as intensity, sets and repetitions. However, this would make the questionnaire longer and more complex. Additionally, research has shown that engaging in muscle-strengthening activities is beneficial, regardless of the specific characteristics of the activities [64]. Indeed, assessing, monitoring, and exploring health-related associations of the frequency and duration of muscle-strengthening activities, with valid and reliable questions at a population level, will be paramount for future updates of the guidelines. The same applies to the questions on balance activities. Although balance activities are usually only mentioned in the older adults’ guidelines [15], these are still important health-related activities across the lifespan. Besides that, the recently created 24-hour movement guidelines for Finland (UKK Institute, 2022), already include balance activities for adults.

### Compliance with guidelines

The QMov24h showed adequate validity (69% to 94.3% of agreement) and reliability (k of 0.38 to 0.87; 80.3% to 94.0% of agreement) results for classifying compliance with the Canadian 24-hour Movement Guidelines. The strategy of having continuous response formats and then categorizing the answers *a posteriori,* is stronger than presenting categorical response options. Indeed, fixed categorical response options (i) do not allow capturing the whole *spectrum* of possible responses, (ii) limit analysing dose-response effects and (iii) compromise further analysis and categorizations. In addition, the use of pre-defined categories as response options may influence how respondents perceive and answer the questions [65, 66]. The QMov24h is adaptable to changes in guidelines, unlike questionnaires with categorical response formats. Of note, a validation study comparing two versions of the questionnaire (one with categorical response format and the other with continuous answering mode) found better results for the continuous answering mode version [62].

### Strengths and limitations

The study’s rigorous methodological approach in developing the QMov24h is a significant strength. The study followed the guidelines of the Consensus-based Standards for the Selection of Health Measurement Instruments (COSMIN) for design and analysis of the QMov24h. This involved a three-stage approach: (i) item construction; (ii) testing face validity with end-users; and (iii) content validity with MovBeh experts. This approach was essential to minimize participant reporting errors by ensuring that questions and instructions were adequately worded for the target population. Another strength of this study is the combination of accelerometry with a contextualized diary for validity and reliability studies.

The questionnaire’s major strengths lie in its development as a response to the shortcomings of existing questionnaires. For instance, it underwent face and content validity assessments, and included questions on the three MovBeh using a 24-hour continuum logic. Additionally, the questionnaire measures LPA, muscle strengthening, and balance activities, which aligns with current guidelines. The QMov24h was designed to be informative, self-explanatory, and visually appealing to minimize recall errors and avoid response bias. Furthermore, the QMov24h demonstrated adequate validity and reliability for each MovBeh and their combination.

This study is not without its limitations. Firstly, the use of a sample with a high proportion of university students (although with a range of participants in terms of age, gender and education); secondly, the use of only one hip-worn accelerometer rather than a combination of accelerometers (hip for PA, thigh for SB and wrist for sleep), recognizing that such a study design would significantly increase the burden on participants. Furthermore, although the hip-worn accelerometer has been shown to be the best option for PA behaviours (mainly MVPA), the thigh-worn accelerometers (e.g. ActivPal) have been shown to be better for posture behaviours (e.g. SB) [67]. For example, evidence suggests that actigraphy promotes overestimation of SB in comparison to ActiPAL on the thigh [68].

## Conclusions

The QMov24h questionnaire is a valid and reliable tool for assessing levels of MovBeh and compliance with guidelines in adults. Its measurement properties are comparable to, or even better than, those of existing questionnaires, while posing a similar burden to participants. The QMov24h is useful for research, clinical practice, and public health surveillance. The QMov24h has strong psychometric properties, making it suitable for translation, cultural adaptation, and testing in diverse populations for broader international use.

## Supplementary Information

Supplementary Material 1.

Supplementary Material 2.

Supplementary Material 3.

Supplementary Material 4.

## Data Availability

The data are available upon reasonable request from the authors.

## Abbreviations

QMov24h: 24-hour Movement Questionnaire
SB: Sedentary
PA: Physical activity
MovBeh: Movement behaviours
IPAQ: International Physical Activity Questionnaire
MVPA: Moderate-to-vigorous physical activity
LPA: Light physical activity
COSMIN: the Consensus-based Standards for the Selection of Health Measurement Instruments
MPA: Moderate physical activity
VPA: Vigorous physical activity
BMI: Body mass index
Cpm: Counts per minute
ICC: Intraclass Correlation Coefficients
CI: Confidence interval
SPSS: Statistical Package for the Social Sciences
LoA: Limits of Agreement
DABQ: Daily Activity Behaviours Questionnaire
STAQ: Sedentary, Transportation and Activity Questionnaire
Min: Minutes
h: Hours
GPAQ: Global Physical Activity Questionnaire
