# Supplementary material for: 24-hour Movement Questionnaire (QMov24h) for adults: development process and measurement properties
Source: Int J Behav Nutr Phys Act. 2024 Oct 9;21:116. doi: 10.1186/s12966-024-01667-7 (PMC11466043; doi:10.1186/s12966-024-01667-7)
Supplement: Supplementary file 1 — Supplementary Material 1. [file 12966_2024_1667_MOESM1_ESM.pdf]

ANNEX 1 - QUESTIONÁRIO 24-HORAS MOVIMENTO (QMOV24H)

24-HOUR MOVEMENT QUESTIONAIRE (QMOV24H)

PROJECT MOVE24

ID ___________

Questionário 24 horas Movimento (QMov24h)

O dia tem 24 horas e durante este tempo podemos estar a **dormir, em comportamento sedentário ou em atividade física.** Nas 24h do dia só podemos fazer **um destes comportamentos de cada vez**. Neste questionário gostaríamos de saber como é que passa o seu tempo habitualmente. Assim, considere, **todos os contextos e momentos em que estes comportamentos ocorrem, seja no trabalho/a estudar, no tempo livre, ou como forma de deslocamento**. Não importa se apenas anda de carro para o trabalho durante 5 minutos, ou se costuma estar sentado/a apenas 10 minutos no autocarro; queremos saber esses pequenos momentos, por favor, tente contabilizá-los todos. Neste questionário consideramos **dias de trabalho/estudo** (normalmente dias de semana) e **dias em que não trabalha** (normalmente ao fim de semana, ou nos dias de folga do trabalho), numa semana típica.

Não existem respostas certas ou erradas. Por favor, responda a cada pergunta de forma precisa e sincera.

Como responder a este questionário?

Vamos perguntar-lhe **quanto tempo passa** a **dormir,** em **comportamento sedentário** e em **atividade física**. Assim, por favor, responda às questões desta forma:

EXEMPLOS:

Imagine que passa 35 minutos num determinado comportamento ou atividade:

Em média, quanto tempo passa ..., por dia?	0 h 35 min, por dia

Imagine que passa 1 hora e 5 minutos num determinado comportamento ou atividade:

Em média, quanto tempo passa ..., por dia?	1 h 05 min, por dia

Dias de trabalho/estudo e dias de folga

Indique por favor, numa semana típica, os dias em que trabalha e os seus dias de folga do trabalho (dias em que não trabalha). Se não tem um padrão de dias de trabalho e de folgas fixo, reporte nesta questão, os dias em que trabalhou e esteve de folga na semana passada.

Se for reformado/a, não responda a esta questão e continue a responder na secção 1.

Se for estudante, considere como dias de trabalho os dias em que estuda ou tem aulas.

	2ª feira	3ª feira	4ª feira	5ª feira	6ª feira	Sábado	Domingo
Dias de trabalho	☐	☐	☐	☐	☐	☐	☐
Dias em que não trabalha (folgas)	☐	☐	☐	☐	☐	☐	☐

ID ___________

Secção 1. Sono Noturno e Sestas

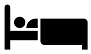

O **sono** noturno é o tempo que dorme durante a noite. Se trabalhar por turnos, ou trabalhar durante a noite, ou se tiver padrões de sono variáveis, tente estimar o número médio de horas no seu período de sono mais longo. As **sestas** são momentos em que dorme durante o dia.

**Numa semana típica, ...**	**num dia de semana ou de trabalho**	**num dia de fim-de-semana ou em que não trabalha**
1. Em média, quanto tempo passa a **dormir** (sono noturno), por dia? *(Não inclua o tempo que passa deitado/a sem estar a dormir)*	____ h _____ min, por dia	____ h _____ min, por dia
2. Em média, quanto tempo passa a fazer a(s) **sesta(s),** por dia?<br>Se não faz sesta(s) assinale com um ✓ aqui ☐	____ h _____ min, por dia	____ h _____ min, por dia

Há quanto tempo mantém este padrão de sono? _______semanas/meses/anos (riscar o que não interessa).

Secção 2. Comportamento Sedentário

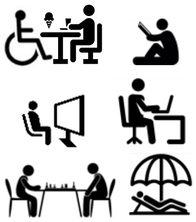

O **comportamento sedentário é o tempo que passa sentado/a, reclinado/a ou deitado/a, enquanto está acordado/a,** em que gasta pouca energia. Durante este tempo pode estar a comer, a ler, a estudar, a ver televisão, a usar o telemóvel, a trabalhar no computador/tablet, a conduzir, sentado nos transportes públicos, na missa, a jogar xadrez, ou noutras atividades, desde que esteja sentado/a, reclinado/a ou deitado/a enquanto acordado.

Nas perguntas seguintes, **contabilize todos os momentos em que está sentado/a, reclinado/a ou deitado/a e acordado/a, desde o momento em que acorda até que adormece.**

**Numa semana típica, ...**	**num dia de semana ou de trabalho**	**num dia de fim-de-semana ou em que não trabalha**
3. Em média, quanto tempo passa **sentado/a, reclinado/a ou deitado/a,** por dia?	____ h _____ min, por dia	____ h _____ min, por dia
3.1. Do tempo que indicou na questão 3, quanto tempo, em média por dia, **passa sentado/a, enquanto trabalha**?	____ h _____ min, por dia	
3.2. Do tempo que indicou na questão 3, quanto tempo, em média por dia, **passa sentado/a, no tempo em que não está a trabalhar**?	____ h _____ min, por dia	

Há quanto tempo mantém este padrão de comportamento sedentário? _______semanas/meses/anos (riscar o que não interessa).

Secção 3. Atividade Física

A atividade física é qualquer movimento que realiza recorrendo aos seus músculos. Existem muitas formas de atividade física, entre as quais:

Aeróbias: atividades em que utiliza os grandes grupos musculares de forma repetida e dinâmica.

(ex.: andar a pé/correr, andar de bicicleta, dançar, jogar futebol ou outro jogo coletivo, etc.)

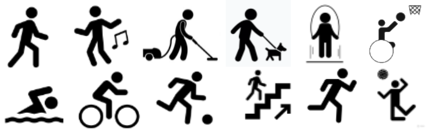

Fortalecimento Muscular: atividades que requerem o uso da força dos músculos.

(ex.: estar de pé, pegar em compras, segurar uma criança ao colo, levantar objetos, arrastar móveis, agachamentos, levantar pesos, exercícios com bandas elásticas ou máquinas de musculação, etc.)

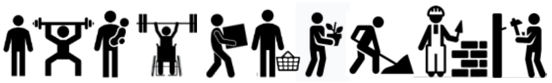

As atividades aeróbias e de fortalecimento muscular podem ser de intensidade ligeira, moderada ou vigorosa:

Atividades Ligeiras

- baixa intensidade
- requerem um esforço mínimo
- batimentos cardíacos ligeiramente superiores aos de repouso
- respiração ligeiramente superior à de repouso
- é possível conversar normalmente
- depois de as realizar não se sente cansado/a

Atividades Moderadas

- intensidade média
- requerem mais esforço do que as atividades ligeiras
- batimentos cardíacos superiores ao das atividades ligeiras
- respiração acelerada
- é possível conversar, mas com alguma dificuldade
- transpira
- depois de as realizar sente-se cansado/a

Atividades Vigorosas

- intensidade elevada
- requerem muito esforço
- batimentos cardíacos superiores ao das atividades moderadas
- respiração ofegante
- não é possível conversar
- transpira mais
- depois de as realizar sente-se muito cansado/a

Nota importante:
A mesma atividade pode requerer esforços de intensidades diferentes em pessoas diferentes. Por exemplo, caminhar pode representar para si um esforço aeróbio mínimo, em que consegue manter uma conversa. Neste caso deve considerá-la de intensidade ligeira. Mas, se caminhar for para si uma atividade muito difícil de realizar, cansando-se e exigindo um esforço muito elevado, deve considerá-la uma atividade aeróbia vigorosa. Numa escala de esforço de 1 a 10, um esforço mínimo é 1 e um esforço máximo é 10:

Atividade ligeira			Atividade moderada			Atividade vigorosa			
1	2	3	4	5	6	7	8	9	10

Esforço mínimo Esforço máximo

Secção 3a. Atividade Física Ligeira

Estime o tempo médio **por dia** em que faz **atividades LIGEIRAS** aeróbias e de fortalecimento muscular.

Numa **semana típica**, ...	**num dia de semana ou de trabalho**	**num dia de fim-de-semana ou em que não trabalha**
4. Em média, quanto tempo passa em **atividade aeróbia ligeira,** por dia?	____ h _____ min, por dia	____ h _____ min, por dia
5. Em média, quanto tempo passa em **atividade de fortalecimento muscular ligeira,** por dia?	____ h _____ min, por dia	____ h _____ min, por dia

Há quanto tempo mantém este padrão de atividade física ligeira? _______semanas/meses/anos (riscar o que não interessa).

Secção 3b. Atividade Física Moderada e Vigorosa

Nas perguntas seguintes, referentes à atividade moderada e vigorosa, pedimos-lhe que estime o tempo em que faz atividade física da respetiva intensidade **em cada um dos dias da semana (semana típica).**

EXEMPLO:

Imagine que faz uma caminhada de 30 minutos à 2ª feira e uma sessão de atividade física de 1 hora à 5ª feira. Suponha que para si essa caminhada representa uma atividade aeróbia moderada. Na sessão de atividade física a intensidade do esforço e o tipo de atividade física podem variar; nestes casos estime o tempo passado em cada tipo e intensidade de atividade. Suponha que durante a sessão de atividade física fez cerca de 5 minutos de aquecimento (esforço aeróbio ligeiro), 25 min em esforço aeróbio moderado, 20 minutos de fortalecimento muscular moderado e 10 min de fortalecimento muscular vigoroso. De acordo com este exemplo deveria responder assim:

	**ATIVIDADES MODERADAS**		**ATIVIDADES VIGOROSAS**	
	**Aeróbias**	**Fortalecimento muscular**	**Aeróbias**	**Fortalecimento muscular**
**2ª feira**	_______ h 25 min	_______ h ______min	_______ h ______min	_______ h ______min
**3ª feira**	_______ h ______min	_______ h ______min	_______ h ______min	_______ h ______min
**4ª feira**	_______ h ______min	_______ h ______min	_______ h ______min	_______ h ______min
**5ª feira**	_______ h 30 min	_______ h 20 min	_______ h ______min	_______ h 10 min
**6ª feira**	_______ h ______min	_______ h ______min	_______ h ______min	_______ h ______min
**Sábado**	_______ h ______min	_______ h ______min	_______ h ______min	_______ h ______min
**Domingo**	_______ h ______min	_______ h ______min	_______ h ______min	_______ h ______min

Estime o tempo médio por cada dia da semana em que faz **atividades de intensidade MODERADA e VIGOROSA aeróbias e de fortalecimento muscular, numa semana típica.**

	ATIVIDADES MODERADAS		ATIVIDADES VIGOROSAS	
	**Aeróbias**	**Fortalecimento muscular**	**Aeróbias**	**Fortalecimento muscular**
**2ª feira**	_______ h ______min	_______ h ______min	_______ h ______min	_______ h ______min
**3ª feira**	_______ h ______min	_______ h ______min	_______ h ______min	_______ h ______min
**4ª feira**	_______ h ______min	_______ h ______min	_______ h ______min	_______ h ______min
**5ª feira**	_______ h ______min	_______ h ______min	_______ h ______min	_______ h ______min
**6ª feira**	_______ h ______min	_______ h ______min	_______ h ______min	_______ h ______min
**Sábado**	_______ h ______min	_______ h ______min	_______ h ______min	_______ h ______min
**Domingo**	_______ h ______min	_______ h ______min	_______ h ______min	_______ h ______min

Se não faz atividades **moderadas** assinale com um ✓ aqui ☐ Se não faz atividades **vigorosas** assinale com um ✓ aqui ☐

Há quanto tempo mantém este padrão de atividade física moderada e vigorosa? _______semanas/meses/anos (riscar o que não interessa).

Secção 3c: Atividades de Equilíbrio

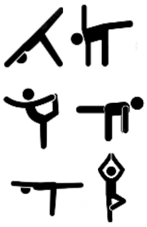

As atividades de equilíbrio são aquelas em que desafia a sua capacidade de se manter estático (sem se mexer) ou que desafia a sua capacidade de equilíbrio enquanto se movimenta, como por exemplo, estar durante alguns segundos apenas com um pé no chão, estar durante alguns segundos parado na ponta dos pés, andar na ponta dos pés, alguns exercícios de Tai Chi Chuan, Yoga ou Pilates, etc.

Estime o tempo médio por cada dia da semana em que faz **atividades de equilíbrio, numa semana típica.**

**2ª feira**	_______ h ______min
**3ª feira**	_______ h ______min
**4ª feira**	_______ h ______min
**5ª feira**	_______ h ______min
**6ª feira**	_______ h ______min
**Sábado**	_______ h ______min
**Domingo**	_______ h ______min

Se não faz atividades de equilíbrio assinale com um ✓ aqui ☐

Há quanto tempo mantém este padrão de atividades de equilíbrio? _______semanas/meses/anos (riscar o que não interessa).

Agradecemos a sua colaboração!
