# Supplementary material for: 24-hour Movement Questionnaire (QMov24h) for adults: development process and measurement properties
Source: Int J Behav Nutr Phys Act. 2024 Oct 9;21:116. doi: 10.1186/s12966-024-01667-7 (PMC11466043; doi:10.1186/s12966-024-01667-7)
Supplement: Supplementary file 2 — Supplementary Material 2. [file 12966_2024_1667_MOESM2_ESM.pdf]

ANNEX 2 - QUESTIONÁRIO 24h MOVIMENTO (QMov24h)

24h Movement Questionaire (QMov24h)

PROJECT MOVE24

ID ____________

24-Hour Movement Questionnaire (QMov24h)

A day has 24 hours, and during this time we can **sleep, engage in sedentary behaviour, or engage in physical activity.** In the 24 hours of a day, we can only **do one of these behaviours at a time.** In this questionnaire we would like to know how you usually spend your time. As such, consider **all contexts and moments when these behaviours occur, whether it is at work/during study, during leisure time, or as a form of commuting**. It doesn't matter if you only drive to work for 5 minutes, or if you only sit for 10 minutes on the bus; we want to know about all these short moments, so please try to account for them all. In this questionnaire we consider **working/studying days** (usually weekdays) and **non-working/studying days** (usually at the weekend, or on days off from work), in a typical week.

There are no right or wrong answers. Please answer each question accurately and truthfully.

How to answer this questionnaire?

We will ask you **how much time** you spend **sleeping**, in **sedentary behaviour**, and in **physical activity**. So please answer the questions in this way:

EXAMPLES:

Imagine you spend 35 minutes on a certain behaviour or activity:

On average, how much time do you spend ..., per day?	_0_ h _35_ min, per day

Imagine you spend 1 hour and 5 minutes on a certain behaviour or activity:

On average, how much time do you spend ..., per day?	_1_ h _05_ min, per day

Working/studying days and non-working days

Please indicate, in a typical week, the days you work and the days off from work (days in which you don't work). If you do not have a fixed pattern of working days and days off, report in this question the days you worked and those you were off from work, during the last week.

If you are retired, do not answer this question, and continue answering in section 1.

If you are a student, consider as working days the days you study or take classes.

	Monday	Tuesday	Wednesday	Thursday	Friday	Saturday	Sunday
Working days	☐	☐	☐	☐	☐	☐	☐
Non-working days (days off)	☐	☐	☐	☐	☐	☐	☐

Block 1. Night-time Sleep and Naps

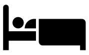

Night-time **sleep** is the time you sleep during the night. If you work on a shift basis or at night, or have variable sleep patterns, try to estimate the average number of hours in your longest sleep period. Naps are periods when you sleep during the day.

**In a <u>typical week</u>, ...**	**on a weekday or on a working day**	**on a weekend day or on a non-working day**
1. On average, how much time do you spend **sleeping** (night-time sleep), per day? *(Do not include the time you spend lying down without sleeping)*	____ h _____ min, per day	____ h _____ min, per day
2. On average, how much time do you spend **napping**, per day?<br>If you don not nap, mark a ✓ here ☐	____ h _____ min, per day	____ h _____ min, per day

For how long have you maintained this sleep pattern? _______weeks/months/years (cross out what does not apply).

Block 2. Sedentary Behaviour

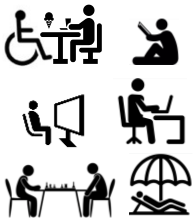

Sedentary behaviour is the time you spend sitting, reclining, or lying down while awake, expending little energy. During this time, you can be eating, reading, studying, watching TV, using a computer/tablet/mobile phone, driving, sitting on public transport, at mass, playing chess, or other activities, as long as you are sitting, reclining or lying down while awake.

In the following questions, **account for all time that you are sitting, reclining, or lying down and awake, from the moment you wake up until you fall asleep**.

**In a <u>typical week</u>, ...**	**on a weekday or on a working day**	**on a weekend day or on a non-working day**
3. On average, how much time do you spend **sitting, reclining, or lying down** per day?	____ h _____ min, per day	____ h _____ min, per day
3.1. Of the time you indicated in question 3, on average, how much time per day, do you **spend sitting down while at work**?	____ h _____ min, per day	
3.2. Of the time you indicated in question 3, on average, how much time per day, do you **spend sitting down when you are not working**?	____ h _____ min, per day	

For how long have you maintained this pattern of sedentary behavior? _______ weeks/months/years (cross out what does not apply).

ID ___________

Block 3. Physical Activity

Physical activity is any movement you perform using your muscles.
There are many forms of physical activity, including:

Aerobics: activities in which you use the large muscle groups repeatedly and dynamically.

(e.g., walking/running, cycling, dancing, playing football or other team sports, etc.)

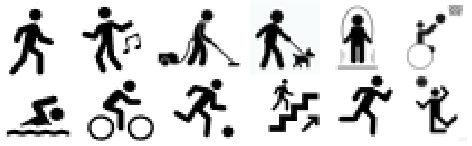

Muscle Strengthening: activities that require the use of muscle strength.

(e.g., standing, picking up groceries, holding a child on your lap, lifting objects, dragging furniture, squats, lifting weights, exercises with elastic bands or weight machines, etc.)

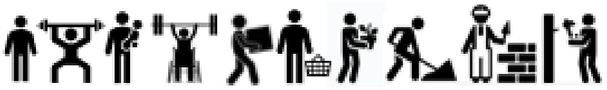

Aerobic and muscle-strengthening activities can be of light, moderate, or vigorous intensity:

Light Activities

- low intensity
- require minimal effort
- heart rate slightly higher than at rest
- breathing slightly higher than at rest
- it is possible to talk normally
- you do not feel tired after performing them

Moderate Activities

- medium intensity
- require more effort than light activities
- heart rate higher than for light activities
- fast breathing
- it is possible to talk, but with some difficulty
- you sweat
- you feel tired after performing them

Vigorous Activities

- high intensity
- require a lot of effort
- heart rate higher than moderate activity
- very fast breathing
- it is not possible to talk
- you sweat more
- you feel very tired after performing them

Important note:
The same activity may require effort of different intensities for different people. For example, walking may represent minimal aerobic effort for you, and you may be able to hold a conversation while walking. In this case, you should consider walking to be of a light intensity. But if walking is a very difficult activity for you to perform because it is tiring and demanding and requires a very high effort, you should consider it a vigorous aerobic activity. On a scale of effort from 1 to 10, a minimum effort is 1 and a maximum effort is 10:

Light Activity			Moderate Activity			Vigorous Activity			
1	2	3	4	5	6	7	8	9	10

Block 3a. Light Physical Activity

Estimate the average amount of time, per day, that you spend doing **LIGHT** aerobic and muscle-strengthening activities.

In a typical week, ...	on a weekday or on a working day	on a weekend day or on a non-working day
4. On average, how much time do you spend in **light aerobic activity**, per day?	____ h _____ min, per day	____ h _____ min, per day
5. On average, how much time do you spend in **light muscle-strengthening activity**, per day?	____ h _____ min, per day	____ h _____ min, per day

For how long have you maintained this pattern of light activity? _______ weeks/months/years (cross out what does not apply).

Block 3b. Moderate and Vigorous Physical Activity

In the following questions concerning **MODERATE and VIGOROUS** activities, we ask you to estimate the average amount of time, for **each day of the week,** you spend doing these intensities **(typical week)**.

EXAMPLE:

Imagine you do a 30-minute walk on Monday and an hour of physical activity on Thursday. Assume that this walk is moderate aerobic activity for you. In the physical activity session, the effort's intensity and the type of physical activity may vary; in these cases, estimate the time spent in each type and intensity of activity. Suppose that during your physical activity session you did about 5 minutes of warm-up (light aerobic activity), 25 minutes of moderate aerobic activity, 20 minutes of moderate muscle-strengthening activity, and 10 minutes of vigorous muscle-strengthening activity. Using this example, you should answer as follows:

	MODERATE ACTIVITIES		VIGOROUS ACTIVITIES	
	**Aerobic**	**Muscle-strengthening**	**Aerobics**	**Muscle-strengthening**
**Monday**	_______ h 25 min	_______ h ______min	_______ h ______min	_______ h ______min
**Tuesday**	_______ h ______min	_______ h ______min	_______ h ______min	_______ h ______min
**Wednesday**	_______ h ______min	_______ h ______min	_______ h ______min	_______ h ______min
**Thursday**	_______ h 30 min	_______ h 20 min	_______ h ______min	_______ h 10 min
**Friday**	_______ h ______min	_______ h ______min	_______ h ______min	_______ h ______min
**Saturday**	_______ h ______min	_______ h ______min	_______ h ______min	_______ h ______min
**Sunday**	_______ h ______min	_______ h ______min	_______ h ______min	_______ h ______min

ID ____________

Estimate the average amount of time, for each day of the week, you spend doing **MODERATE and VIGOROUS aerobic and muscle-strengthening activities in a typical week**.

	**MODERATE ACTIVITIES** 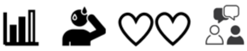		**VIGOROUS ACTIVITIES** 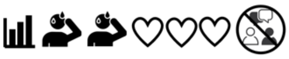	
	**Aerobics**	**Muscle-strengthening**	**Aerobics**	**Muscle-strengthening**
**Monday**	_______ h ______min	_______ h ______min	_______ h ______min	_______ h ______min
**Tuesday**	_______ h ______min	_______ h ______min	_______ h ______min	_______ h ______min
**Wednesday**	_______ h ______min	_______ h ______min	_______ h ______min	_______ h ______min
**Thursday**	_______ h ______min	_______ h ______min	_______ h ______min	_______ h ______min
**Friday**	_______ h ______min	_______ h ______min	_______ h ______min	_______ h ______min
**Saturday**	_______ h ______min	_______ h ______min	_______ h ______min	_______ h ______min
**Sunday**	_______ h ______min	_______ h ______min	_______ h ______min	_______ h ______min

If you do not engage in **moderate** activities, mark with a ✓ here ☐ If you do not engage in **vigorous** activities, mark with a ✓ here ☐

For how long have you maintained this pattern of moderate and vigorous activities? _______ weeks/months/years (cross out what does not apply).

Block 3c: Balance Activities

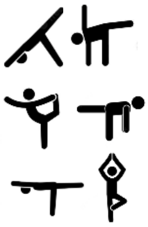

Balance activities are those in which you challenge your ability to remain static (without moving) or those which challenge your ability to balance while moving, such as standing for a few seconds with only one foot on the floor, standing on tiptoes for a few seconds, walking on tiptoes, and some exercises from Tai Chi Chuan, Yoga or Pilates, etc.

Estimate the average amount of time, for each day of the week, you spend doing **balance activities, in a typical week**.

**Monday**	_______ h ______min
**Tuesday**	_______ h ______min
**Wednesday**	_______ h ______min
**Thursday**	_______ h ______min
**Friday**	_______ h ______min
**Saturday**	_______ h ______min
**Sunday**	_______ h ______min

If you do not engage in balance activities, mark with a ✓ here ☐

For how long have you maintained this pattern of balance activities? _______ weeks/months/years (cross out what does not apply).

Thank you for your co-operation!
