# Supplementary material for: 24-hour Movement Questionnaire (QMov24h) for adults: development process and measurement properties
Source: Int J Behav Nutr Phys Act. 2024 Oct 9;21:116. doi: 10.1186/s12966-024-01667-7 (PMC11466043; doi:10.1186/s12966-024-01667-7)
Supplement: Supplementary file 4 — Supplementary Material 4. [file 12966_2024_1667_MOESM4_ESM.pdf]

Annex 4

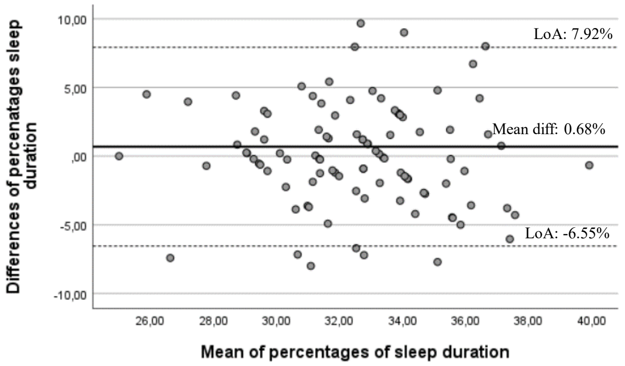

Fig.1a. Bland-Altman plot for percentage of time spent in sleep per 24h. The difference between objectively measured and self-reported time spent in sleep. Mean difference: 0.68 ± 3.69% of 24h (p=0.515); LoA: -6.55 to 7.92h/day.

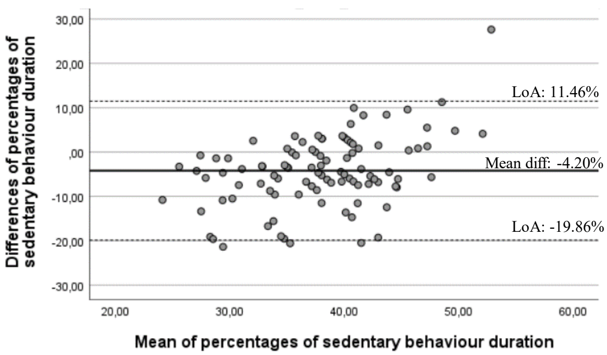

Fig.1b. Bland-Altman plot for percentage of time spent in sedentary behaviour per 24h. The difference between objectively measured and self-reported time spent in SB. Mean difference: -4.20 ± 7.99% of 24h (p≤0.001); LoA: -19.86 to 11.46%.

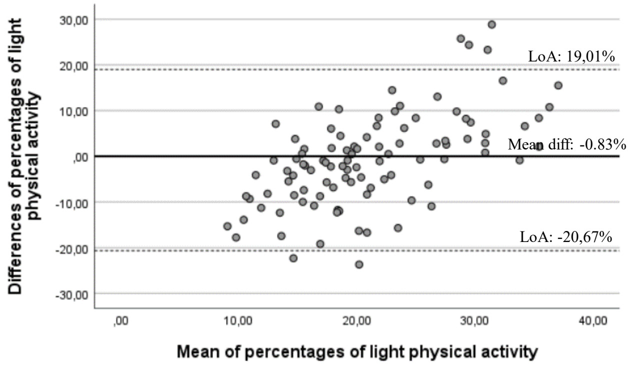

Fig.1c. Bland-Altman plot for percentage of time spent in light physical activity per 24h. The difference between objectively measured and self-reported time spent in LPA. Mean difference: -0.83 ± 10.12% (p≤0.001); LoA: -20.67 to 19.01%.

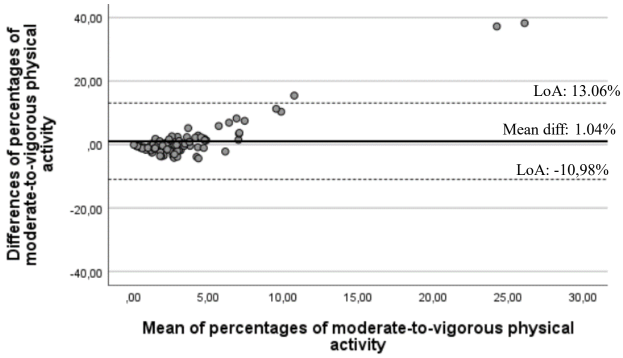

Fig.1d. Bland-Altman plot for percentage of time spent in aerobic MVPA per 24h. The difference between objectively measured and self-reported time spent in aerobic MVPA. Mean difference: 1.04 ± 6.13% (p≤0.001); LoA: -10.98 to 13.05%.

Figure 1. Bland-Altman plot with MovBeh expressed as proportions of 24-hour
